# A Rare Case of Post-lumbar Discectomy Pneumocephalus: An Anatomically Informed Case Report

**DOI:** 10.7759/cureus.81147

**Published:** 2025-03-25

**Authors:** Yasir H Elhassan, Mustafa Alhasan, Yasser S Abdulghani

**Affiliations:** 1 Basic Medical Sciences, Taibah University, Madinah, SAU; 2 Neuroradiology, Taibah University, Madinah, SAU; 3 Surgery, The National Ribat University, Khartoum, SDN

**Keywords:** cerebrospinal fluid leak, clinical neuroanatomy, dural tear, lumbar discectomy, pneumocephalus, spinal surgery complications

## Abstract

Pneumocephalus is an uncommon yet significant complication that can arise after lumbar discectomy and requires rapid diagnosis and intervention. Although cerebrospinal fluid (CSF) leakage is not frequently observed during these procedures, it can result from small, often inconspicuous dural defects that may be missed during surgery. This scenario underscores the importance of careful intraoperative inspection and vigilant postoperative monitoring to ensure timely recognition and management, thereby mitigating potential adverse outcomes.

A 36-year-old male patient underwent an L5-S1 lumbar discectomy for disc herniation. Eight days postoperatively, he developed severe headache, neck pain, and nausea, accompanied by CSF leakage confirmed through beta-2 transferrin testing. Computed tomography revealed significant pneumocephalus, while high-resolution MRI demonstrated a subtle dural defect. Despite no macroscopically visible dural tear during re-exploration, applying an epidural blood patch successfully resolved both the CSF leak and pneumocephalus.

This case underscores the value of advanced imaging, specifically high‐resolution MRI, in identifying subtle dural defects that may not be apparent during surgery. Detecting the minor CSF leakage was essential for directing subsequent management and avoiding further complications.

Early recognition of postoperative symptoms combined with comprehensive imaging assessment is crucial in the management of post-discectomy pneumocephalus. The rapid resolution observed after applying an epidural blood patch supports its role as a primary intervention strategy, even in cases where dural tears are not macroscopically evident.

## Introduction

Lumbar discectomy represents one of the most frequently performed spinal surgical procedures, primarily aimed at addressing intervertebral disc herniation and associated neurological symptoms [1]. While generally considered safe and effective, this intervention carries inherent risks, including the potential for cerebrospinal fluid (CSF) leakage [2]. The rate of CSF leaks after primary lumbar discectomy has been estimated to be 3.5%, while the rate of leaks after revision surgery is 13.2% [1]. Nonetheless, the occurrence of pneumocephalus, especially as it is a direct complication of lumbar discectomy, is infrequent and not always reported in the literature [3-5].

When it occurs, pneumocephalus can contribute to significant morbidity. The morbidity is multifaceted and largely depends on the extent of intracranial air (ICA) accumulation and whether the air causes a “tension” phenomenon leading to increased intracranial pressure (ICP). In mild cases, patients may experience persistent headaches and altered sensorium, while more severe presentations can include a range of symptoms such as nausea, vomiting, confusion, and, in rare instances, seizures or even focal neurological deficits [2,3,5].

The anatomical basis of the pneumocephalus is the interrelationship with the cranial compartments, the ventricular system, the subarachnoid space, and the brain. The consequences of ICA are classic for pathological changes in the CSF spaces and alteration of the bodily compartments in the rigid cranial vault, significantly when the role of pressure is changed by spinal procedures [6]. However, the anatomical considerations do not end with the surgical site; they continue up the cerebrospinal axis and membranes [5]. This is a valuable approach for both the development and the management of pneumocephalus as it is.

To understand ICP dynamics, one must understand the structures involved: the cranium, ventricular system, subarachnoid space, brain tissue, and the vascular system. Under normal circumstances, CSF is secreted in the ventricles and passes through the third and fourth ventricles to the subarachnoid space to surround the brain [7,8]. In a normal person, the intracranial cavity has a tight fit of brain, CSF, and blood.

The theory by Monro and Kellie states that the intracranial cavity has a fixed volume, which means that any increase in one component, such as ICA, must be accompanied by a decrease in another [9]. When ICA increases in size, it pushes on the CSF and blood, which may open the path to increased ICP [10]. This integrated anatomical analysis also helps to explain the pathogenesis of pneumocephalus and highlights the role of careful preoperative planning and close postoperative care in the prevention and management of this uncommon adverse event.

## Case presentation

**Figure 1:**
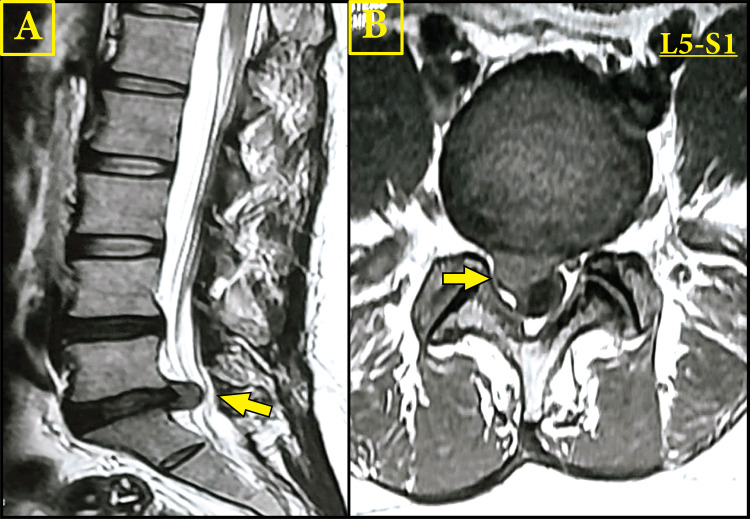
An MRI scan of the lumbar spine Magnetic resonance imaging demonstrated an L5-S1 moderate-sized right-sided subarticular disc extrusion compressing the traversing nerve roots on the right side of the spinal canal. A: T2 sagittal MRI shows the lumbar spine with an arrow indicating a disc herniation; B: T1 axial (cross-sectional) MRI view of the L5-S1 level, with the arrow pointing to the corresponding herniated disc material.

A 36-year-old male soldier with no history of trauma or other chronic illnesses presented with severe lower back pain radiating to the right lower extremity, significantly restricting his movement and interfering with his activities of daily living (ADLs). Clinical examination revealed intact motor strength (5/5) in all muscle groups, normal sensory function, and symmetric deep tendon reflexes. The straight leg raising test was positive at 30° on the right side, and his pain intensity was rated 8/10 on the Visual Analog Scale (VAS). Magnetic resonance imaging demonstrated an L5-S1 moderate-sized right-sided subarticular disc extrusion compressing the traversing nerve roots on the right side of the spinal canal, as presented in Figure 1. The combination of a high VAS score, the impact on ADLs, a positive clinical examination, and corroborative imaging findings supported the decision to proceed with surgical intervention.

The patient underwent standard lumbar microdiscectomy under general anesthesia. The surgical procedure involved a midline incision at the L5-S1 level, followed by muscle dissection, lamina exposure, limited laminotomy, and microsurgical discectomy. No apparent dural tear was noted during the procedure. Subsequently, the Valsalva maneuver was performed to exclude any potential CSF leakage, and routine closure was performed in layers [11].

**Figure 2:**
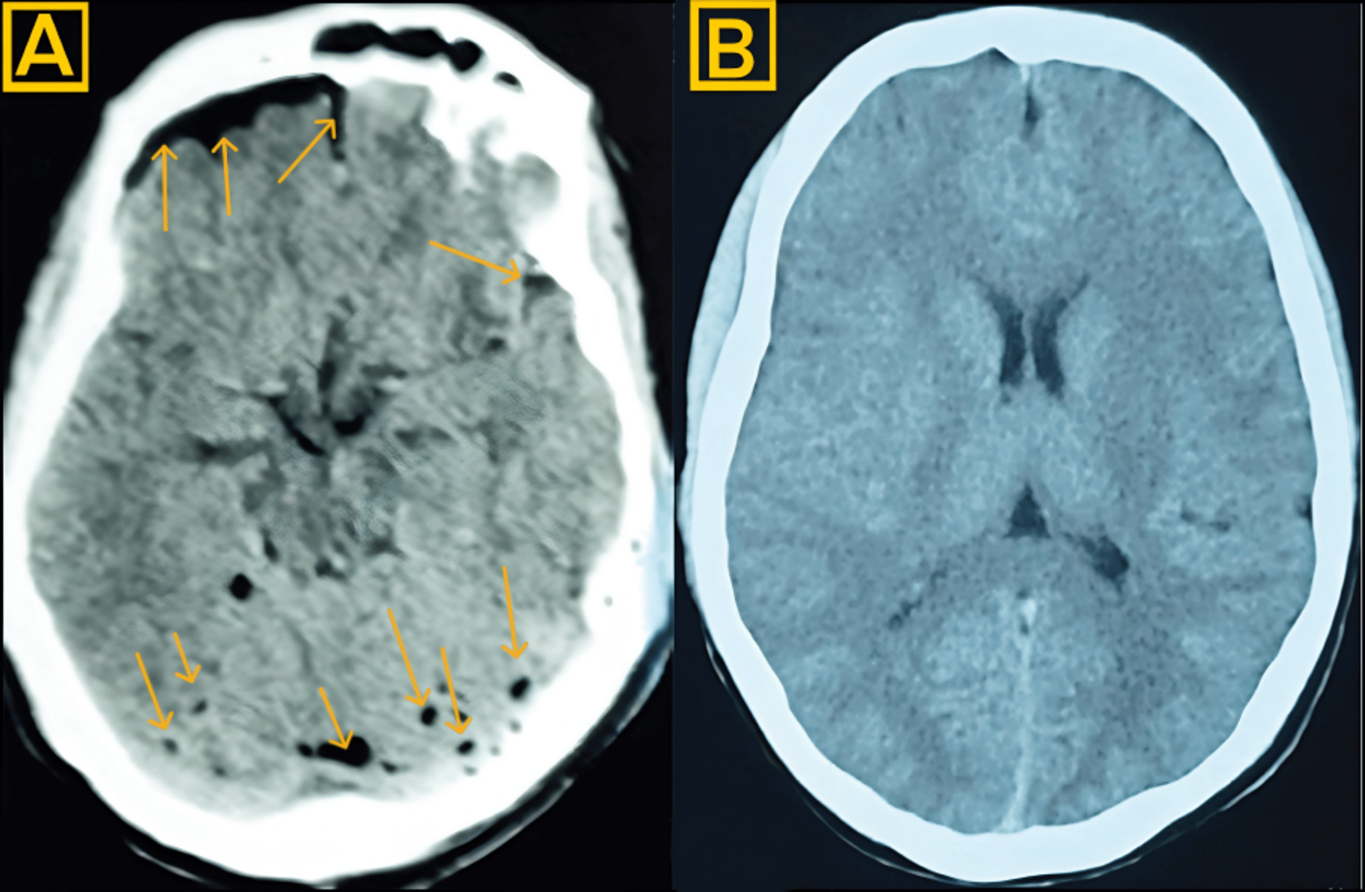
Axial non-contrast images of the brain CT (A) Initial CT demonstrated significant pneumocephalus (illustrated by arrows) on postoperative day eight; (B) Follow-up CT after epidural blood patch showing resolution of intracranial air collections.

The initial recovery period progressed without incident until postoperative day eight when the patient developed severe positional headache (VAS 9/10), neck pain and stiffness, persistent nausea and vomiting, and clear fluid leakage from the surgical site. Beta-2 transferrin testing confirmed the presence of CSF in the wound drainage. Computed tomography demonstrated significant frontal pneumocephalus without midline shift or hemorrhage (Figure 2A).

**Figure 3:**
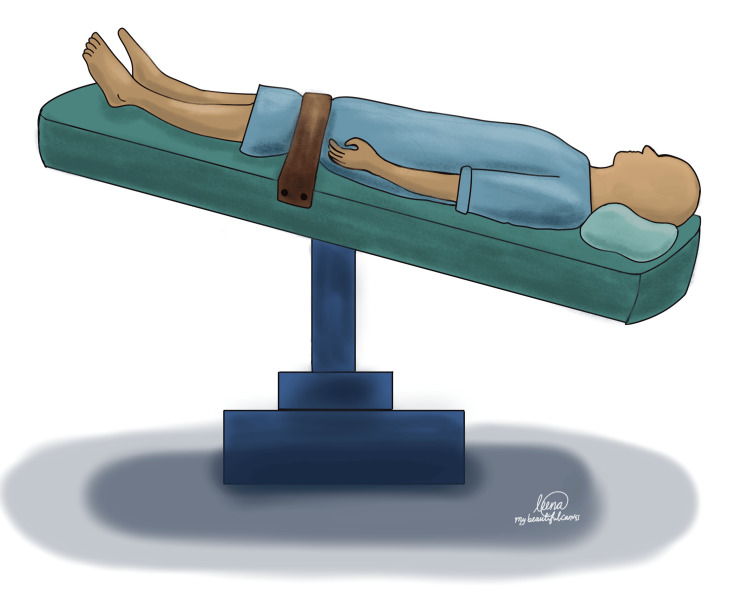
Trendelenburg position (15 to 30 degree incline) Trendelenburg position was utilized in the conservative management of pneumocephalus. This positioning helps redistribute intracranial air and potentially alleviates symptoms. Image credits: Created by Dr. Liena Babiker Mekki using Procreate; permission for use granted.

Initial conservative management was initiated with 30° Trendelenburg positioning (Figure 3), intravenous hydration, prophylactic antibiotics, bed rest, and oral caffeine supplementation.

**Figure 4:**
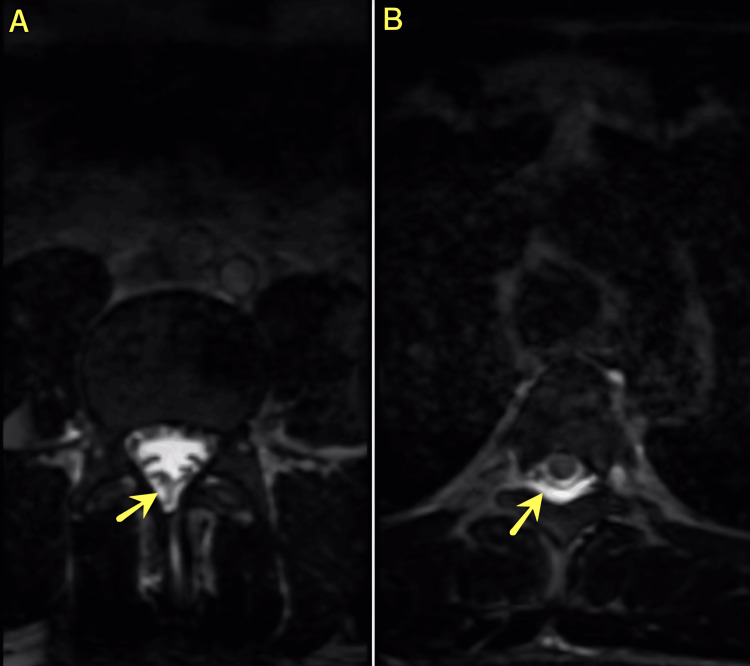
Axial high-resolution T2-weighted MRI demonstrating the dural defect and associated CSF leakage. (A) A small dural defect is visualized along the right dorsal aspect of the lumbar thecal sac at the level of L5-S1. (B) A T2 hyperintense collection is seen along the dorsal aspect of the thoracic thecal sac, consistent with a CSF fluid leak. CSF: cerebrospinal fluid

Due to persistent symptoms, a high-resolution MRI was obtained, which demonstrated a subtle dural defect accompanied by evidence of CSF leakage along the right dorsal aspect of the lumbar thecal sac at the L5-S1 level (Figure 4A) and with hyperintense collection along the dorsal aspect of the thoracic thecal sac, indicative of active CSF leakage (Figure 4B). Subsequently, surgical re-exploration was performed; however, no macroscopic dural tear was identified intraoperatively. Based on the imaging findings, an epidural blood patch was applied using 15 mL of autologous blood, followed by layered closure with additional fascial reinforcement. The patient experienced complete resolution of symptoms within 72 hours, and follow-up CT confirmed the resolution of pneumocephalus (Figure 2B).

## Discussion

This paper presents a rare case of pneumocephalus following a post-lumbar discectomy, which illustrates the difficulties of CSF leaks after spinal surgeries that can have severe neurological consequences if undiagnosed and untreated.

The patient had typical symptoms of post-operative headache, neck pain, and fluid discharge from the surgical site. Establishing the CSF leakage diagnosis by beta-2 transferrin assay was important for further surgical planning [12]. Nevertheless, although there was no evident dural defect during the initial surgery or the subsequent re-exploration, the high-resolution MRI showed a minor dural defect (Figure 4). This is in accordance with the study by Patel et al. [3], which showed that dural defects may not be easily noticed during surgery and that other imaging techniques should be used for diagnosis.

The approaches to treating complications after discectomy are not always consistent and well-defined; however, this report presents a practical approach to using an epidural blood patch to treat a complication. Previous studies have shown that epidural blood patches effectively block minor dural defects and rehabilitate CSF homeostasis [11].

**Figure 5:**
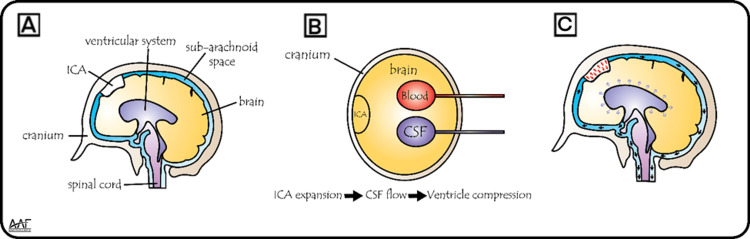
Anatomical basis of intracranial hydrodynamics in pneumocephalus (A) Sagittal schematic illustrating the cranial vault, ventricular system, subarachnoid space, and continuation into the spinal canal, highlighting the pathway of cerebrospinal fluid (CSF) and the potential for intracranial air (ICA) entry; (B) Axial view depicting the three principal intracranial components, namely, brain tissue, CSF, and blood, with an additional illustration of ICA, whose expansion necessitates compensatory volume shifts in accordance with the Monro-Kellie doctrine; (C) Diagram demonstrating the effects of ICA expansion, including the displacement of CSF and blood, which may contribute to elevated intracranial pressure. Image credits: Created by Ayman A. Fayek using Adobe Illustrator; permission for use granted.

Pneumocephalus following lumbar discectomy is a pathophysiological process through the ‘reverse bottle’ effect and the ‘ball-valve’ mechanism when a unidirectional valve allows air to enter the cranial cavity but not exit [10,13]. In our case, the imaging studies' symptoms and findings can be related to the formation of a negative pressure gradient due to CSF loss, as mentioned in the literature. Also, the interaction dynamics between the intracranial air and the CSF flow depicted in our anatomical framework (Figure 5) and other works clearly illustrate the importance of balance within the cranial cavity [9].

Also, the measures that were initially adopted in the patient's management included positioning and hydration, which were very important in relieving symptoms before surgery. The efficacy of such conservative measures has been noted in other case reports of similar conditions of pneumocephalus, where the patient was positioned in the Trendelenburg position (15-30-degree incline) to help redistribute ICA and potentially alleviate symptoms; it was found to be a crucial part of the management plan (Figure 3) [14].

Recommendations and limitations

In patients with postoperative symptoms suggestive of CSF leakage, who have a challenging diagnosis of subtle dural defects that may not be evident intraoperatively, early use of advanced imaging modalities such as high-resolution MRI is recommended. However, when conservative measures fail, the epidural blood patch can be very effective, as it was in this case.

The major limitation of this report is that it is based on a single-case design and may not be generalizable to all patients undergoing lumbar discectomy. Further research is needed to elaborate on the incidence, optimal diagnostic methods, and management strategies for post-discectomy pneumocephalus, which will be addressed in future work involving larger case series and standardized imaging protocols.

## Conclusions

In conclusion, this case indicates the need for early diagnosis and management of complications following lumbar discectomy, including pneumocephalus. As shown by the present study and the literature review, the use of advanced imaging methods is crucial for detecting minor dural defects that may not be noticed during the surgery. This paper has also illustrated how using an epidural blood patch effectively treated this case of CSF leak, even in the absence of obvious dural defects. In the future, we propose to increase the stringency of imaging protocols and preventive surgical practices in order to prevent such complications and improve the quality of life of patients, as well as the knowledge about the treatment of post-discectomy complications.
